# Whole Medical Systems versus the System of Conventional Biomedicine: A Critical, Narrative Review of Similarities, Differences, and Factors That Promote the Integration Process

**DOI:** 10.1155/2017/4904930

**Published:** 2017-07-13

**Authors:** Erik W. Baars, Harald J. Hamre

**Affiliations:** ^1^European Scientific Cooperative on Anthroposophic Medicinal Products (ESCAMP), Zechenweg 6, 79111 Freiburg, Germany; ^2^Louis Bolk Institute, Hoofdstraat 24, 3972 LA Driebergen, Netherlands; ^3^University of Applied Sciences Leiden, Zernikedreef 11, 2333 CK Leiden, Netherlands; ^4^Institute for Applied Epistemology and Medical Methodology, Witten/Herdecke University, Zechenweg 6, 79111 Freiburg, Germany; ^5^Witten/Herdecke University, Gerhard-Kienle-Weg 4, 58313 Herdecke, Germany

## Abstract

**Background:**

There is an increasing need for a worldwide professional integration of conventional medicine and traditional/complementary whole medical systems (WMSs). However, the integration is perceived by conventional medicine as problematic or unacceptable, because of a supposed lack of evidence for specific effects of WMSs therapies and supposed prescientific or unscientific paradigms of WMSs.

**Objectives:**

To review the literature on the features of WMSs, similarities and differences between conventional medicine and WMSs, and scientific and clinical practice issues that should be dealt with in order to promote the integration process.

**Methods:**

A critical, narrative review of the literature on six WMSs.

**Results and Conclusions:**

Key factors for the integration of WMSs and conventional medicine are as follows: legal frameworks, quality standards, high-quality research on safety and efficacy of WMS interventions, infrastructure, and financial resources. For scientific assessment of WMSs, there are unresolved ontological, epistemological, and methodological issues and issues of diagnostics, therapy delivery, and outcome assessment in clinical practice. Future research not only should be directed at quality assurance and generating the necessary data on safety and efficacy/effectiveness but also should address more fundamental (ontological, epistemological, and methodological) issues, in order to overcome the differences between WMSs and conventional medicine.

## 1. Introduction

“Medicine is a science and practice of intervention, manipulation, and control concerned with curing sick people, caring for sick people, preventing maladies, and promoting health” [1] (p. IX). Throughout human history, different cultures in all parts of the world have had their own type of medicine. In Western countries and cultures, conventional, biomedical-based medicine has been developed, rooted in the natural sciences that had developed since the Middle Ages, [2]. In many non-Western cultures but also in Western cultures, several types of whole medical systems (WMSs) [3], that is, complete systems of theory and practice that have evolved independently over time in different cultures and apart from conventional medicine or Western medicine [3], have been developed.

Currently WMSs, often referred to as traditional and complementary medicine (T&CM) or (traditional and) Complementary and Alternative medicine (TCAM/CAM), and conventional medicine are found in almost all countries in the world. WMSs are in increasing demand by patients and are also studied in universities (e.g., the Academic Consortium for Integrative Medicine & Health in the USA). According to the “Traditional Medicine Strategy: 2014–2023” of the World Health Organization (WHO), “the public and consumers of health care worldwide continue to include T&CM in their health choices. This obliges Member States to support them in making informed decisions about their options” [4] (p. 18). “As the uptake of T&CM increases, there is a need for its closer integration into health systems. Policy makers and consumers should consider how T&CM may improve patient experience and population health” [4] (p. 19). A central argument in favor of integrating T&CM into conventional medicine is that T&CM has additional knowledge and interventions on preventive and curative health promotion [5]. The integration can therefore contribute to current issues in public health and healthcare such as developing strategies of healthy ageing, promoting self-management, and controlling healthcare expenditures [6, 7]. Positive examples demonstrating and supporting the WHO strategy of integrating the best of both of worlds from T&CM and conventional medicine are the integration of the AYUSH (Ayurveda, Yoga, Naturopathy, Unani, Siddha, and Homeopathy (AYUSH)) system in the conventional system in India [8], the increasing use of mindfulness techniques in the treatment of depressive disorders [9], and the use of traditional medicinal systems in reducing the prescription of antibiotics in Thailand as one of the strategies to fight the global antimicrobial resistance problem [10].

This WHO position on integration of T&CM and conventional medicine is in contrast with the developments in many Western countries. Whereas many traditional medical systems were tolerated in clinical practice next to or integrated with conventional medicine in many Western countries until the end of the 20th century, this situation has rapidly changed, as a result of two interrelated developments regarding science-based medicine:

- The first development is the increasing dominance of evidence-based medicine (EBM) in medicine since the 1990s [11]. As a result, ideally, only therapies with high-quality scientific evidence (from systematic reviews and meta-analyses of randomized controlled trials) on safety and (cost) effects are accepted in medicine [12]. And although in practice many conventional medical guidelines for a large part are based on lower quality scientific evidence (including clinical expertise), for opponents of the integration of T&CM and conventional medicine, a lack of high-quality scientific evidence is often used as an argument against integration.
- The second development has to do with the roots of science in theory development and theory testing [13]. In the last decades, the dominance of the biomedical model in medicine has led to scientific criticism of WMSs due to their theoretical basis that is perceived as not being in agreement with biomedical theories, but based on paradigms deemed as prescientific or unscientific. Furthermore, there is allegedly no evidence for specific effects of CAM medicinal products for conventional indications as tested in clinical studies according to the EBM paradigm [14, 15]. And although in science currently the reductionist model is increasingly challenged and the WMSs theoretical models appear to be content-wise in line with the systems approaches in science and medicine, opponents of the integration of T&CM and conventional medicine use the assumed prescientific or unscientific theoretical models of WMSs often as an argument against integration.

On the other hand, there are positive examples of integration, like the integrated use of Ayurveda and conventional medicine in treating elephantiasis in India, that resulted in a lifetime achievement award from the International Society of Dermatologists for Oxford professor of Dermatology Terence Ryan; the highly successful integrated treatment of depression with Yoga medicine and conventional medicine in the National Institute of Mental Health and Neuroscience in Bangalore (Bengaluru) in India; and the widely adopted use of Yoga medicine for geriatrics in Japan.

Given the current need for some type of integration of conventional medicine and traditional/complementary medicine in countries all over the world, the large amount of scientific publications and the ongoing scientific debate on this topic among proponents and opponents, we decided to perform a critical review on the literature. Our aim is to provide a transparent overview on similarities and differences between WMSs and the conventional medical system and, based on this overview, identify issues that have to be dealt with in order to overcome the differences. It is expected that this overview will support informed decision-making in the integration process.

## 2. Material and Methods

### 2.1. Research Questions

A critical, narrative review of the literature was performed on the following research questions:

- *In order to describe the domain of WMS*, what are the historical, clinical practice, ontological, epistemological and methodological aspects of WMSs?
- *In order to clarify whether a uniform approach or diverse approaches to their integration are most appropriate*, what are the major similarities and differences between the different WMSs?
- *In order to clarify the generalizability of experiences from integrating non-WMS CAM therapies with conventional medicine*, what are the major differences between WMSs and other “single component” or non-WMS CAM therapies?
- *In order to demonstrate the common grounds and issues to overcome in the integration process*, what are the similarities and differences between WMSs and conventional medicine?
- What are the consequences for the testing of effects of medicinal products (MPs) from WMS (WMPs) and for regulation of WMPs?
- *In order to facilitate the integration process*, which aspects need attention to promote the integration of conventional medical system and the WMSs?

### 2.2. Scope of the Review, Databases, and Search Terms

In order to address the six research questions, we included the following six WMSs: Traditional Chinese Medicine (TCM) [16, 17], Ayurveda [18, 19], Unani Medicine [20], Homeopathy [21], Naturopathy [22], and Anthroposophic Medicine (AM) [23, 24]. A discussion of “all” WMSs around the world was beyond the scope of the paper; the selection includes WMSs established in four large populations/cultures (China, Indian subcontinent, Arabic/Muslim countries, and Western cultures).

Beyond the scope of this article is a comprehensive review of the discussions of the topics of (supposed) lack of evidence on specific effects of WMS treatments and the (supposed) lack of tested WMS theories. Nonetheless, these topics are briefly discussed.

We searched the database PubMed, Google Scholar, and our own literature archives. Combinations of search terms used were as follows: whole medical systems, TCM, Ayurveda, Unani, Homeopathy, Naturopathy, or AM in combination with features, philosophy, methodology, or ontology.

## 3. Results

### 3.1. Overview and Historical Development of the Whole Medical Systems

A condensed overview of the WMSs included in this review is presented in Table 1. Of these six WMSs, three (TCM, Ayurveda, and Unani) are based on old traditions (first classical texts in the first millennium BC, predated by oral transmissions from the second millennium BC), each from a specific culture: TCM developed in China in connection with the philosophical traditions of Taoism (Lao Tzu, 605–531 BC) and Confucianism (Confucius: 551–479 BC), with classical TCM texts written in the period 221–207 BC [25, 26]. Ayurveda developed on the Indian subcontinent in connection with Hinduism; classical Ayurveda texts are variously dated c700–200 BC [27, 28]. Unani has roots in Greek medicine (Hippocrates: 460–370 BC; Dioscorides: 40–90 AD; Galen: 130–210 AD); a seminal classical text from mainstream Medieval medicine and still used in Unani is the Canon of Medicine (1025 AD) by Ibn Sina (Avicenna, 980–1037 AD) [29]. These three WMSs have existed in their respective cultures for millennia before the development of natural science-based, conventional medicine [3, 16, 17, 19, 21–23, 30].

Three other WMSs (Homeopathy, Naturopathy, and Anthroposophic Medicine) are comparatively younger (<250 years), although traces of influences by older traditions can be found [31–33]. Seminal publications appeared for Homeopathy in 1796 [34], for Naturopathy in 1848 [35], and for AM in 1925 [36]. These three WMSs were first practiced in Central Europe by medical doctors and were further developed next to conventional medicine within Western cultures.

In the course of globalization, all six WMSs have become disseminated from their original culture into other countries and regions, sometimes with establishment of “second centers” such as for Naturopathy in North America [37] and Homeopathy in India [38]. Currently, in almost every country in the world, one or more types of WMS are practiced [4].

### 3.2. Similarities and Differences

#### 3.2.1. Similarities and Differences between the WMSs

Some major* similarities *between the WMSs are as follows:

- Holistic, nonatomistic ontological, epistemological, and practice orientation
- Aiming at preventive as well as curative health promotion
- Individualized treatment based on a system approach
- Medicinal use of a large number of different substances and WMPs, of mostly herbal but also mineral and zoological origin (e.g., 700 herbal species in TCM [39], >1000 substances in homeopathy [40], >4000 herbal species in Ayurveda, and >800 substances in AM [41])
- Nonmedication treatment modalities including massage, physical exercises, hydrotherapy, thermotherapy, and diet (although each modality may be applied differently, cf. Table 1)

Some major* differences* between the WMSs are as follows:

- Use of different languages, including different concepts of levels of wholeness
- Different diagnostic systems
- Different specific therapy modalities, for example, acupuncture in WMS and art therapies in AM

Homeopathy has two particular aspects:

- In the development of homeopathy, there have been a strong element of pure empiricism and relatively less emphasis on theory.
- Homeopathic diagnostics and treatment are usually limited to case taking and the prescription of homeopathic MPs [42]. All homeopathic MPs are manufactured according to specific homeopathic procedures such as potentization, that is, successive dilution, each dilution step involving a rhythmic succession (repeated shaking of liquids) or trituration (grinding of solids into lactose monohydrate). In contrast, treatment in the other five WMSs is to a much larger extent multimodal (Table 1).

A particular aspect of Naturopathy is the widespread use of therapy modalities from other WMSs (e.g., Chinese herbs and homeopathic MPs) or from non-WMS CAM (e.g., food supplements) [43]. In contrast, the other WMSs have a stronger element of uniformity, either in their theory (TCM, Ayurveda, Unani, and AM) or in the use of one specific type of WMPs (Homeopathy).

TCM and Ayurveda have long traditions of mainly oral transmission of WMS knowledge and experience, predating the classical texts [16, 26].

Particular aspects of AM include the broad spectrum of artistic therapies deployed (painting, clay modeling, drawing, recitation, and music exercises) and the use of AM treatments also in large hospitals offering accident and emergency service within public requirement plans [23]. WMPs used in AM can be manufactured according to specific anthroposophic methods or methods used for herbal, homeopathic, or conventional MPs [41].

#### 3.2.2. WMSs versus Other “Single Component” or Non-WMS CAM Therapies

The major differences between WMSs and single component CAM interventions are as follows:

- Some single component (or fixed combination of) CAM interventions can be conceptualized within the conventional biomedical paradigm: for example, a number of vitamins are used as CAM therapy, while their purported pharmacological effects are conceptualized on the levels of cell biology or biochemistry. In contrast, WMS interventions are not so easily understood on these levels (although the difference is not absolute [5]).
- Single component CAM interventions can be protocolled for specific conventional and/or CAM indications, whereas this is not the case for WMS interventions.
- Individualized, multimodal CAM treatment entails the combination of several treatment modalities that are tailored to the needs of the individual patient. When this happens within a WMS, all treatment modalities are understood within and derived from one conceptual framework, leading to a uniform treatment approach. When diverse single component CAM interventions are combined, a uniform conceptual understanding is often not possible, leading to eclecticism.

#### 3.2.3. Similarities and Differences between WMSs and the System of Conventional Biomedicine

The main* similarities* between WMSs and some developments in conventional medicine are as follows:

- The development of a personalized medicine/individualization approach in addition to the current mainstream protocolled approach [44, 45]
- The use and role of professional judgment in some domains of clinical practice (e.g., interpretation of radiographs) [45, 46]
- The increasing use of complex interventions [47–49]
- System approaches in diagnostics and therapy (e.g., systems biology, epigenetics, emergentism, metabolomics, “network medicine,” “polypharmacology,” and “polytarget treatment”) [5, 50–52]
- Shared decision-making [45, 53]
- A holistic dynamic health concept [5, 54]
- The use of pattern recognition methodologies [55, 56]
- The notion that RCTs are not applicable everywhere [57, 58] with a shift towards more pragmatic trials [45, 48, 58, 59] and other study types [48, 60]
- The notion that conducting clinical studies for multiple clinical conditions and their respective diverse therapy options has its limitations, due to excessive complexity and prohibitive costs
- The increasing role of patient preferences and patient autonomy
- The real-world situation that, in many medical fields (e.g., paediatric surgery, emergency medicine, and vaccination), RCT-based practice is only marginal and often critically questioned

The main* differences* between the WMSs and the conventional medicine system are summarized in Table 2 [61].

### 3.3. Integration of WMSs and the Conventional Medicine System

The integration of WMSs and conventional medicine entails some key, interdependent factors:

- Legislation: therapy providers and WMPs
- Education: practitioners of the two integrating medical systems that have to work together for a period of years to build up experience and confidence in effective team work
- Quality standards for WMS treatment: training of providers, delivery of treatment, and pharmaceutical quality of WMPs
- Scientific research on the safety and efficacy of WMP interventions
- Infrastructure and financial resources

Regarding scientific research and quality benchmarking, there are specific issues pertaining to the inherent properties of WMSs:

- Ontological, epistemological, and methodological issues relevant for the overall understanding and assessment of WMSs
- Specific issues relevant for diagnostics, therapy delivery, and outcome assessment in clinical practice

These issues are discussed in the following subsections.

#### 3.3.1. Legislation, Quality Standards, Research, Infrastructures, and Resources

Of paramount importance for integration is the recognition of WMSs in* legislation*, in particular

- recognition of WMS therapy providers, their training schools and diplomas;
- regulatory provisions enabling the registration or marketing authorisation of WMPs.

Such recognition is dependent on establishing* quality standards*:

- For therapy providers (e.g., WHO benchmarks for training in TCM [26], Ayurveda [62], Unani Medicine [63], and Naturopathy [33]; CEN [French: Comité Européen de Normalisation] standards for health care provision by medical doctors with additional qualification in Homeopathy [64])
- For the pharmaceutical quality of WMPs (e.g., the Anthroposophic Pharmaceutical Codex [41]).

Scientific and societal recognition also depends on high-quality evidence for efficacy/effectiveness and safety of WMPs and nonmedication treatment in WMSs; hence, integration also includes the funding, conduct, and publication of* scientific research* studies in order to generate and disseminate such evidence. In order to promote quality standards and scientific research,* infrastructures* and* financial resources* are needed.

In some countries these tasks are given national priority: for example, the Government of India supports research, education, quality standardization, and infrastructure building for seven WMSs (AYUSH: Ayurveda, Yoga, Naturopathy, Unani, Siddha, Homeopathy, and Sowa-Rigpa), since 2004 within the newly established Ministry of AYUSH [65].

One example of infrastructure building is establishing Integrative Medicine centers in academic hospitals, where specific WMS modalities (not necessarily the entire WMS) are developed, applied, and tested. This model has been implemented in the USA and organized in the Academic Consortium for Integrative Medicine & Health, based on four pillars: (1) the horizontal relationship between the doctor/therapist (coach) and the patient (coproducer); (2) the active role of the patient in prevention (lifestyle), wellbeing, and therapy and healing processes; (3) the use of evidence-based safe and effective conventional and complementary therapies; and (4) the use of healing environments [66].

#### 3.3.2. Fundamental Ontological Aspects

All studied WMSs take a* nonatomistic, holistic ontological *position towards the nature of reality. This means that they all conceptualize, each in a different form, in addition to material elements and forces, the existence of nonmaterial forces working in nature and man, which also play a role in health and disease. For example, a central concept of TCM is Qi, a vital energy or life force that moves in the body through a system of pathways called meridians [16]. Similar concepts are found in Ayurveda (“prana” [67]) and Unani (“arwah” or vital spirit [29]). AM has the concept of four levels of formative forces working in man: formative physical forces and three nonmaterial forces (life, soul, and spirit) [23, 30]. Homeopathy conceptualizes the nonmaterial effects of high potentized substances [21] and also Naturopathy is based on holistic and vitalistic principles [22].

#### 3.3.3. Conceptual and Epistemological Aspects

In line with the nonatomistic, holistic ontological position, central concepts of WMS are holistic. Concepts of the human being emphasize the wholeness and complexity of the human being [68]; its emergent, nonlinear dynamic, and epigenetic properties; and its ability of self-organization and adaptation as a network system [69]. Health is conceptualized as the ability to balance and actively restore the wholeness of the human being [5]. Within the WMS practice methods, there is an essential role for intuition and expert knowledge in diagnostics and decision-making [45], while treatment also takes into account context factors and the uniqueness, constitution, and complexity of the individual [45, 70]. In conceptualizing causality, WMSs emphasize systems causality [71], effects that involve global and patterned shifts across multiple subsystems of the person as a whole, and the role of context/placebo and intention effects [69].

#### 3.3.4. Methodological Aspects

A review on clinical and epidemiological research in CAM [48] demonstrates that, for research on therapy effects, there is consensus that both efficacy and effectiveness studies have their own place, validity, and importance. Some authors argue that efficacy research should be prioritised over effectiveness research to legitimise the use of CAM and to help to increase acceptance. Other authors state that efficacy research to examine specific effects should not be undertaken until overall effectiveness of the therapy in question is demonstrated, in order to prevent misuse of scarce resources. This discussion also reflects different opinions on the importance and value of specific and nonspecific effects within the whole of clinical practice. An integrative research approach has been described as simultaneous research into mechanisms and overall effectiveness of CAM treatments. Contemporary methodological standards of medical research can be applied to CAM research, but it might be necessary to adapt the research designs in some areas, in order to account for the complexity of CAM interventions [72]. CAM-specific challenges must be addressed, such as the problem of strict standardization of diverse treatments and study participants leading to lack of external validity. RCTs do not answer all research questions and are expensive to conduct. Placebo-controlled RCTs might be inappropriate for some specific CAM modalities. There is a need for additional methods, for example, pragmatic studies [73], observational studies, mix of qualitative and quantitative studies, and *n* = 1 studies.

In treatment studies, there is on the one hand the tendency to operationalize WMS interventions into a “treatment package” that can be used also outside the original WMS context, and on the other hand the critique that some essential aspects (e.g., individualization) or therapy components may become excluded by such operationalization, leading to reduced efficacy and misperceptions of the “true” traditional WMS intervention [74].

Outcomes should be broader than symptom reduction alone, they should contain several levels of the whole human being, including physical, mental, spiritual, and social factors [70]. Health economic evaluation of CAM treatments was seen as particularly relevant in modern healthcare. Research into the mechanisms of placebo, context, or meaning effects were also seen as important to determine appropriate control groups and their respective explanatory power, in order to explain potentially contradictory study results and to maximize these effects in clinical practice. Newer evaluation models such as program theory, the theory on “the mechanisms that mediate between the delivery (and receipt) of the program and the emergence of the outcomes of interest” [75, 76], encompass a wide range of health-related changes that include process aspects, such as the emergence of new meanings and understanding during or after treatment, as well as longer term changes in health, wellbeing, and health-related competences and behavior [77].

Another proposed model is a “reversed research strategy” for assessing CAM, starting with studies of the context, paradigms, philosophical understanding, and utilization, then subsequently the safety status of the whole system, comparative effectiveness of the whole system, and specific efficacy of components, and finally the underlying biological mechanisms [49, 78]. Other, expressly nonhierarchical models include a circular information synthesis of different evidence forms [45, 60] and an “evidence house” [79].

#### 3.3.5. Clinical Practice Aspects

Main topics with regard to WMS practice methods pertain to the development of whole system diagnostics and interventions; the development and application of quality control systems for individualized diagnostics and treatment and the use of multidisciplinary complex interventions [80]; the role of protocols, guidelines, and expert knowledge in clinical practices of a whole system approach [81]; and the use of double (conventional and WMS) diagnoses.

A WMS diagnosis is a diagnosis on the level of the individual patient and is system-based. In practices where WMS is integrated with conventional medicine, we therefore find double diagnoses. Diagnostics on the individual and system level often includes pattern recognition methods which require interrelated expert knowledge, intuition, and system thinking skills [82].

In WMS therapy the focus is on the sick patient in his or her whole complexity, including physical, mental, spiritual, and social factors. These are interconnected and need to be addressed in total and on multiple levels. The repertoire of CAM treatment is often multimodal and complex, and its application highly individualized. CAM treatments and counselling are provided as integrative systems with interacting components. Accordingly, the effects of complex approaches are often larger than the sum of the components' effects. WMS therapy aims to support and stimulate autoprotective and salutogenetic potentials (self-healing and self-regulatory abilities), mostly with the active cooperation of the patient or of his/her organism. WMS practices also require a good patient-practitioner interaction (therapeutic relationship) and cocreation of the patient in varying therapeutic contexts [45]. Clinical evaluation includes patient-determined outcomes as well as patient satisfaction [70]; notably, these outcome measures are also becoming increasingly used in evaluation of conventional treatments.

#### 3.3.6. Quality and Clinical Safety of WMPs

Regarding the pharmaceutical quality and clinical safety of WMPs, there is a difference in the historical development of the older and newer WMSs.

In the 20th century, homeopathic and anthroposophic MPs have been marketed in European countries such as Austria, France, Germany, and Switzerland as drugs, manufactured according to Good Manufacturing Practice standards, and subject to modern drug regulation including pharmacovigilance. Toxicologically relevant starting materials (e.g., aconite and cinnabar) are highly diluted according to safety requirements of European regulations [83]. Adverse reactions to these MPs are infrequent and usually of mild to moderate severity; anaphylactic reactions occur but are very rare [40, 84, 85].

In contrast, MPs from Chinese, Ayurveda, and Unani medicine have historically been produced for local use. In modern times, industrial-scale production has developed with less rigorous quality control, and MPs have been regulated as food or food supplements or have been imported for use without regulation. Some WMPs have been associated with repeated, severe adverse reactions, including liver and kidney toxicity (sometimes fatal) [86–88], heavy metal poisoning [89–93], epileptic seizures [94], and adrenal suppression from undeclared addition of corticosteroids to herbal products [90]. There are further concerns regarding environmental contaminations (e.g., air pollution, soil contaminations), cultivation practices (e.g., pesticides, fungicides, microorganisms, endotoxins), manufacturing procedures (e.g., microorganisms, endotoxins), and inappropriate use [95, 96]. In order to overcome these problems, pharmacovigilance systems have been established in the main producing countries of Chinese, Ayurvedic, and Unani MPs [97, 98], and there are considerable efforts to improve the quality standards for these WMPs [99, 100].

### 3.4. Mismatches and Aspects Needing Attention

#### 3.4.1. Mismatches

Currently, based on the described differences, there are mismatches between the current scientific empirical (EBM) and theoretical (biomedical model) demands and the properties and specificity of WMSs. We describe these mismatches here by means of the example of WMPs, with the demands and their application in drug regulation on the one hand and WMPs and the inherent properties of WMS on the other hand. The main mismatches are as follows:

1. WMPs are insufficiently tested because they are not in line with conventional interests and biomedical models.
2. WMPs are generally handled as standardized conventional medicinal product (CMP) interventions, whereas they should also be handled as part of a complex intervention.
3. WMPs are handled as CMPs, that is, symptom reducing, fighting disease therapy, whereas they should be handled as a curative, health promotion therapy that supports the self-healing abilities of the organism.
4. WMPs are tested for conventional indications based on group-oriented taxonomy and diagnostics, whereas they should be tested for individualized WMP indications.
5. WMPs are assumed to have specific biochemical effects like CMPs have, but WMP therapy is directed at higher levels, aiming at the regulation and harmonizing (e.g., Dosha balancing in Ayurveda) of overarching, complex physiological processes, and the transformation of physiological and psychological processes and capacities into more mature and integrated states (Schad, 2008; Simon, 2009).
6. WMPs are often judged on efficacy by regulatory authorities as new CMPs, whereas they should also be regarded as part of a traditional WMS with long-standing use, developed following a reverse pathway compared to CMPs (Fønnebø et al., 2007; Kienle et al., 2011).

The main consequences of these mismatches are as follows:

1. The dominance of the biomedical model has resulted in an a priori negative image and rejection of WMPs by scientists of conventional biomedicine, whereby, seen from a reductionist, mechanistic position, effects of WMPs are regarded as mere nonspecific, context effects, not worthy of serious scientific scrutiny.
2. As a consequence of this attitude of rejection, there is an underrepresentation of WMS scientists in academic institutions and scarce public funding of academic WMP research.
3. Many WMPs are not tested in clinical research and can therefore not obtain ordinary marketing authorisation.
4. WMPs are most often not tested according to their theoretical higher order, system level effects but are tested in conventional RCTs with a single product approach. Therefore the precision of the tested WMP treatment is decreased, with an increased high risk of “false-negative results” (meaning: in reality the treatment has beneficial effects but these are not captured in the research study).
5. WMPs often do not appear in guidelines for treatment of specific conventional indications since many WMPs are not in line with mainstream biomedical theories, are not tested in clinical research, and are not part of the expert knowledge of the developers of conventional treatment guidelines.

This development is not restricted to WMPs: there is an increasing call for excluding all WMS modalities from healthcare and for stopping the development of Integrative Medicine, since many WMS interventions are perceived to lack a plausible scientific efficacy model and because relevant results of clinical studies are lacking, for reasons described above.

However, as described previously, there are also positive examples of the integration of WMSs and conventional medicine in practice, examples of high-quality evidence of specific effects of WMS treatment for conventional indications, and WMSs theoretical models that appear to be content-wise in line with the systems approach in science and medicine (see Introduction).

#### 3.4.2. Aspects That Need Attention to Promote the Integration of the Conventional Medical System and the WMSs

From this overview of the differences between the conventional medical system and the WMSs, a number of issues can be deduced that should be dealt with in science and clinical and regulatory practice, in order to overcome the differences and facilitate the integration processes of the best of both worlds.

- Ontological issues
  - Future research and scientific discussion should focus on the nature of reality (matter, organism, mind,…), the tenability of the nonatomistic holistic position of WMSs within the so-called holism-reductionism debate, and ontological issues to overcome in the integration process.
- Conceptual and epistemological issues
  - Future research and scientific discussion should focus on developing and testing theories that are system- and complexity-oriented and that are compatible with both WMS and conventional medicine.
  - Specific theories that conceptually may bridge the two approaches should be further studied: theories of health, disease, healing [101]; individualization in diagnostics and treatment; and health promotion.
- Methodological issues
  - Future use of research methodologies/designs should focus on
    1. a “reversed research strategy” for assessing CAM;
    2. taking into account the complexity of CAM interventions and the role of expert knowledge, intuition, and individualization of diagnostics and therapies;
    3. the health economic evaluation of CAM treatments;
    4. the mechanisms of placebo, context, or meaning effects [102].
- Clinical practice issues
  - Future development and implementation of integrative treatment approaches should take into account
    1. alternatives for protocols and guidelines that are in line with the holistic and individualizing treatment approaches;
    2. the integrated use of dual diagnoses (from both systems);
    3. the integrated use of analytical and system thinking [18];
    4. the optimal integration of “fighting disease” and “health promotion” treatment options.
    
- Regulatory issues
  - Regulatory frameworks must be modified in order to match the specific features of WMPs.
  - New conceptualizations regarding benefit-risk assessment, research synthesis from different types of evidence (not just RCTs), and the evaluation of WMPs are needed. This in line with the opinion of the EU commission that has acknowledged the need for appropriate regulation also of WMPs [103].

#### 3.4.3. Lack of Evidence of Specific Treatment Effects and Prescientific or Unscientific Theories

Whereas the main argument of many people from conventional medicine is that integration of WMSs and conventional medicine is unacceptable due to an assumed lack of evidence of specific effects from WMS treatment and because of alleged prescientific or unscientific theories, we here discuss these issues in more detail.

Apart from the fact that there are some good quality studies demonstrating specific effects of a single WMS therapy for a conventional indication [104], the described features of WMS approaches demonstrate that WMS therapy most often is aiming at system effects and at restoring balances rather than symptom reduction and often contains different treatments as part of a complex intervention. This situation makes it often difficult to test one single, protocol-based treatment for a conventional indication. If this type of evidence would be mandatory, the precision of the tested treatment would be decreased, with an increased high risk of “false-negative outcomes” (meaning: in reality the treatment has beneficial effects but these are not captured in the research study). In addition, it would lead to a feasibility bias against WMSs. This was the reason for the development of the previously described different “reversed research strategy” for assessing CAM [49] and the model of nonhierarchical, circular information synthesis of different evidence forms [60].

With regard to the theories, WMSs theories are (nonatomistic) holistic and not reductionistic and therefore often regarded as prescientific or unscientific. However, the current situation is that throughout different fields of research, scientists increasingly question the ability of pure reductionist theories to describe and explain the complexity of biological organizations [51]. Therefore, new theories (e.g., systems biology, emergence, and epigenetics) originating from the research fields of the biological complexity in organisms and the genome project demonstrate a shift from reductionist towards more holistic concepts. [21] To our opinion, based on these shifts in science, more openness and acceptance towards (nonatomistic) holistic theories is warranted.

## 4. Discussion

There is an increasing need for a worldwide professional integration of conventional medicine and traditional/complementary whole medical systems. However, in many Western countries, the integration is perceived by conventional medicine as problematic and not acceptable. We therefore reviewed the literature on the features of WMSs, the similarities and differences between conventional medicine and WMSs, and future scientific and clinical practice issues that should be dealt with in order to promote the integration process.

Key factors for the integration of WMSs and conventional medicine are as follows: legal and regulatory frameworks for therapy providers and WMPs; quality standards for the training of therapy providers, the delivery of treatment, and the pharmaceutical quality of WMPs; high-quality scientific research on the safety and efficacy of WMS interventions; and adequate infrastructure and financial resources in order to carry out these tasks.

For scientific research and quality benchmarking, there are fundamental issues pertaining to the inherent properties of WMSs: ontological, epistemological, and methodological issues relevant to the overall understanding and assessment and issues relevant to diagnostics, therapy delivery, and outcome assessment in clinical practice. Many of these issues are as yet unresolved, with contradictory positions among scientists and stakeholders of conventional biomedicine and WMSs, respectively, and with mismatches for resource allocation and drug regulation.

The main contribution of this article is that it will provide (more) overview and clarity on this topic for both WMSs and conventional medicine. It will give objective input for rational discussions on the integration topic. In addition, it will support organizations in their preparation and decision-making during the integration process.

A limitation of the article is that we did not include all WMSs, for example, Yoga medicine, osteopathy, Campo, or WMSs from Africa or South America. Also we did not employ all possible search terms, for example, Ayurvedic (in addition to Ayurveda). A topic that is beyond the scope of this article is that we did not discuss the (supposed) lack of evidence on specific effects of WMS treatments and the (supposed) lack of tested theories of WMSs [15] in depth. However, we described the fundamental (ontological, epistemological, and methodological) underlying differences between WMSs and conventional medicine that are related to these issues (evidence of specific effects and lack of tested theories) and made clear why both sides have different perceptions on these issues.

Future research activities not only should be directed at the “forefront issues” of quality assurance and generating the necessary data on safety and efficacy/effectiveness of WMS interventions but also should address the more fundamental (ontological, epistemological, and methodological) issues, in order to overcome the differences between WMSs and conventional medicine.

## Figures and Tables

**Table 1:** Overview of six whole medical systems.

	Chinese	Ayurveda	Unani	Homeopathy	Naturopathy	Anthroposophic
Classical texts	221–207 BC	Variously dated c700–200 BC	Avicenna, 1025 AD	Hahnemann, 1796	Gleich, 1848	Steiner & Wegman, 1925
Use (main regions)	East Asia	South Asia	South Asia, Middle East	Europe, India	Europe, English-speaking countries	Europe, South America
Key concepts	2 forces (yin, yang), vital energy (qi), 5 elements, meridians	3 energies or doshas (pitta, vaka, kapha), 5 elements	7 naturals (elements, temperaments, humors, organs, forces, actions, spirit)	Like cures like, minimum dose, experiments in healthy persons	Healing power of nature, treat the cause of disease, do no harm, doctor as teacher, treat the whole person, prevention	4 levels of formative forces (physical, life, soul, spirit), 3-fold constitution (nerve-sense, rhythmic, motor-metabolic)
Medicinal products & substances	Herbs, minerals, zoological	Herbs, minerals, zoological	Herbs, oils, perfumes	Homeopathic^*∗*^	Herbs, homeopathic^*∗*^, Chinese, food supplements	Homeopathic^*∗*^, herbs, minerals, zoological, chemically defined
Massage	Tuina, shiatsu	Ayurveda massage	Tadlik massage		Swedish massage	Rhythmical massage
Physical therapy		Hydrotherapy	Hydrotherapy, thermotherapy		Hydrotherapy, thermotherapy, joint manipulation	Hydrotherapy, thermotherapy, external applications
Other nonmedication treatment	Acupuncture, moxibustion	Purgation, lifestyle counselling	Purgation, cupping, leeching	Case taking, lifestyle counselling	Acupuncture, lifestyle counselling	Artistic (music, speech, painting, drawing, clay), biography & lifestyle counselling
Physical exercises	Qigong, Tai-Chi	Breathing	Yes	Yes	Yes	Eurythmy movements
Other self-treatment		Heliotherapy, meditation	Reciting sacred text	Stress reduction	Heliotherapy, relaxation techniques	Meditation
Diet	Yes	Yes	Yes	Yes	Yes	Yes

^*∗*^Homeopathic medicinal products can be of herbal, mineral, or zoological origin or chemically defined and are defined by specific homeopathic manufacturing procedures (see text).

**Table 2:** Differences between conventional medicine and whole medical systems (WMSs).

	Conventional medicine	WMSs
Worldview/philosophy	Biomedical/humanistic model	Holistic/spiritual/bio-psycho-spiritual-social model

Health	Default situation of the machine	Result of self-regulating inner activity (e.g., of the organism or psychosocial)
		Restoring wholeness/balance
		(Re)establishment of the harmony between the functions of body, soul and spirit

Disease	Breakdown of the machine	Expression of system imbalance and/or insufficiency of the wholeness creating forces
	Deviance from biological norms	Disequilibrium between biological, psychological, social and spiritual forces
	Has no intrinsic meaning	Entails a potential for human development

Diagnosis	Group level (often, not always)	Individual level
		System level

Treatment	Group-oriented guidelines/protocols (often)	Complex individualized interventions
	Fighting disease	Health promotion
	Requires external resources	Requires internal resources/body, mind and spirit are interrelated and must all be considered in healing
	Use of pharmacotherapy with predominantly specific effects and high use of technology	Use of WMS pharmacotherapy and nonmedicinal therapies with system effects
